# A multi-layer map-oriented resource organization system for web-based self-directed learning combined with community-based learning

**DOI:** 10.1186/s41039-015-0012-2

**Published:** 2015-07-22

**Authors:** Hangyu Li, Shinobu Hasegawa, Akihiro Kashihara

**Affiliations:** 1 School of Information Science, Japan Advanced Institute of Science and Technology, 1-1, Asahidai, Nomi, Ishikawa 923-1292 Japan; 2 Center for Graduate Education Initiative, Japan Advanced Institute of Science and Technology, 1-1, Asahidai, Nomi, Ishikawa 923-1292 Japan; 3 Graduate School of Informatics and Engineering, The University of Electro-Communications, 1-5-1, Chofugaoka, Chofu, Tokyo 182-8585 Japan

**Keywords:** Web-based learning, Self-directed learning, Resource organization, Topic maps, Multi-layer map model

## Abstract

The main issue addressed in this paper is how to improve the learning situation of self-directed learning in resource search and organization from the web. In this paper, we have firstly proposed a multi-layer map model that visualizes basic learning behaviors when using the web for locating and organizing learning resources. It provides learners with the structures of the found resources, the tools for their semantic management, and also a simplified method to share the resources via the map representation. A system based on the proposed model has also been developed, that enables individual learners to easily locate suitable learning resources from the web by referring resource maps and also to organize them as personal topic maps. As community-based learning, by referring to a community topic map which merges all the personal topic maps created by individual self-directed learners, the learners can share their own resources and select those of other learners into their learning topics. As a result, the learners re-organize their personal topic maps by taking the resources from the community topic maps and at the same time contribute to the community topic map through their personal topic maps. A case study conducted to evaluate the effectiveness of the system showed several positive results which validated our proposal.

## Background

In order to enrich one’s knowledge repository, people need to conduct self-directed learning constantly. With the occurrence of the World-Wide Web, accessing to needed information has become easiest ever. From that time, the information loaded on the web has been growing exponentially along with the constant rise of internet technologies.

Therefore, it has been believed that the needed information can be accessed on the web conveniently. Consequently, it has become possible to overcome the restrictions of time and space for self-directed learning which has been demonstrated to enhance the learning process (Thuering et al. 1995), but often requires learners not only to navigate web resources to construct knowledge learned from the resources but also to control the navigation and knowledge construction processes (Schnackenberg et al. 1998; Kashihara and Hasegawa 2005; Hasegawa and Kashihara 2006). As a result, web-based self-directed learning has become an important research area in the past decade. In order to address this issue, our approach is to integrate self-directed learning into community-based learning through which the learners are able to have informal community-centered communications (Fujimoto et al. 2006; Farooq et al. 2007). Community-based learning also attracts attention along with the rapid growth of the web technology. In particular, there are number of researches on social bookmarking which indicate that the community-based learning resources organized by community members with a similar learning interest are expected to be valuable and effective (Millen et al. 2007; Noll and Meinel 2007). However, it is difficult for the learners to access suitable learning resources from community-based learning since the learning goals vary from learner to learner, which leads to the necessity of proper recommendation for community learning resources. In order to address this problem, we have designed the proposed model, the Multi-layer Map Model (Li and Hasegawa 2010) based on an ISO standard named Topic Maps (ISO/IEC 13250 2002). This model enables the learners to visualize common learning behaviors employed on the web, such as locating learning resources, categorizing found resources, and sharing the resources among community members. We have proposed a resource organization system (Li et al. 2012) which connects web contents and learning topics by means of multi-layer map visualization. A case study intended to determine whether the learners could improve the efficiency of their self-directed learning was conducted to assess the effectiveness of this system (Li et al. 2013). After analysis of the experiment data, some encouraging conclusions were drawn which indicated that through topic map representations provided by the system, learners were able to locate appropriate learning resources faster, organize learning resources in a more meaningful way, and collect learning resources inside their learning community more easily and effectively.

## Issue addressed

### Self-directed learning

**Fig. 1:**
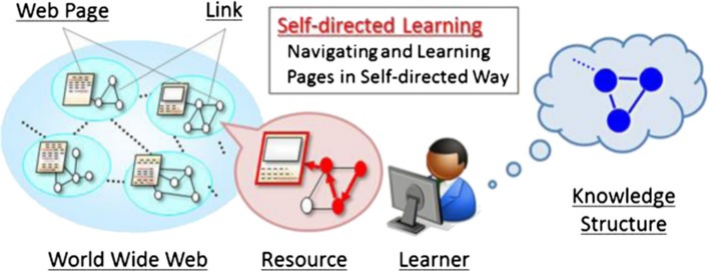
Self-directed learning

Knowles (1975) described self-directed learning as a process in which individuals take the initiative, with or without the help of others, to diagnose their learning needs, formulate learning goals, identify the learning resources, select and implement learning strategies, and evaluate learning outcomes. For identifying learning resources nowadays, learners can navigate a vast volume of web-based resources to achieve their individual learning goals. Such resources usually provide them with hyperspace which enables them to navigate in a self-directed way by following links among the pages as shown in Fig. 1. Self-directed learning is expected to enhance their information literacy by encouraging the selection of suitable resources, each of which may have a different credibility and/or viewpoint of the same topic (Hasegawa et al. 2003; Dabbagh and Kitsantas 2004).

### Community-based learning

**Fig. 2:**
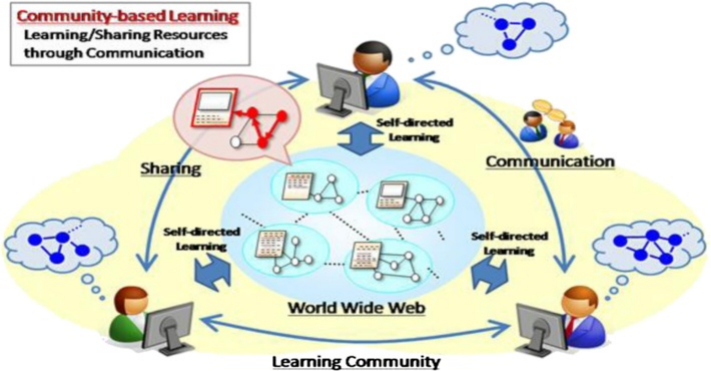
Community-based learning

In this paper, community-based learning is defined as the process of communication by community members who share the similar learning goals for the purpose of encouraging each other’s self-directed learning activity. Figure 2 shows the process that involves not only sharing resources but also performing peer review of the resources found.

Ordinarily, it is not so easy for self-directed learners to obtain adequate supports since the learning resources and the processes vary from learner to learner (Ota et al. 2005). However, community-based learning makes it possible for the learners to engage in informal communication as feedback in their individual self-directed learning processes (Cook and Smith 2004).

### Difficulties in self-directed and community-based learning

The large amount of information available on the web makes it very difficult for the learners to locate suitable learning resources for particular topics of interests. They may have experienced the tedious job of trying to find a link out of pages of listings triggered by Google. Even in some websites exclusively designed for learning, the numbers of pages are so large that it normally takes a learner so much time to find his/her needed information. Traditional search engines only generate lists of pages ranked according to a matching algorithm. The learners therefore often have to click into certain web pages to find out whether they are appropriate or not to achieve their learning goals, and may miss the opportunity to learn if, after two or three useless clicks, they give up. If the learners do finally successfully locate sufficient learning resources from several URLs as a learning hyperspace, they have to organize these resources and to construct their knowledge by navigating the hyperspace. Inexperienced self-directed learners sometimes lose sight of their learning goals because of the complexity of the hyperspace. Such navigation problems have been recognized as major issues, and have been discussed in the context of educational hypermedia/hypertext system development (Brusilovsky 1996). It has indeed become easiest ever to find like-minded people as community members on the web, and the learning resources organized by them seem more reliable and beneficial to self-directed learners since they share the same learning interests, the benefit of which has been proved more than once by social bookmarking (Carmel et al. 2010). However, from the perspective of community-based learning which, from the point of view of this paper, means people with similar learning interests who are willing to review and share learning information on the similar learning topics, it is difficult to pass on learning resources and get feedback among members, for redundancy of learning information is hard to detect, and the viewpoints of each community member are often different.

### Related work

As web-based self-directed learning has become more and more eye-catching, attention from many researchers are being drawn. Being aware of the fact that it is difficult to provide adaptive learning resources to self-directed learners, Pythagoras and Demetrios (2005) introduced a methodology which generated all possible learning paths while matching the learning goals, enabling the learners to select the desired resources from the paths proposed; on the other hand, Kashihara et al. (2002) proposed a similar approach of providing the learners with the adaptive preview of a sequence of web pages as potential navigation path. Dragan and Marek (2006) adopted a different method of mapping ontology for the improvement for resource searching from a semantic web. For resource management, there were tools for constructing local indexes for learning resources found from the web (Hasegawa et al. 2003), in which a framework for reorganizing existing web-based learning resources with indexes representing their characteristics was designed, which consist of “How To Learn” indexes and “What To Learn” indexes, in order to build a learning resource database. As for community-based learning, the learning opportunities of social bookmarking service have also been discussed (Liu and Chang 2008).

Although these researches relating to web-based learning have greatly enhanced the learning situation on the web from various points of view, they either targeted an enclosed learning environment, or certain educational hypermedia which involved not only the learner but also the instructor. Meanwhile, the basic learning behaviors of web-based self-directed learning usually occur in procession, but these research only focused on one or two learning situations and did not take into consideration the seamless combination of learning activities such as resource finding and organization.

Concept map (Novak and Gowin 1984) and knowledge map (O’Donnell et al. 2002) are diagrams that represent ideas as node-link assemblies which has been prevalently studied in many researches. Back in the late 90s, Dansereau and Newbernm (1997) pointed out that semantic displays, such as knowledge maps, were becoming more prevalent in educational settings, and an experiment conducted by Chmielewski and Dansereau (1998) indicated that training participants on the construction and use of knowledge maps made participants recall more macro and micro level ideas from text passages than those without taking the training. Not only in educational setting but in learning contexts, there were also researches proving the concept/knowledge map to be more effective for attaining knowledge retention and transfer than reading text-based learning contents (McCagg and Dansereau 1991; John and Olusola 2006), and more beneficial working as navigational aids than a contents list (McDonald and Stevenson 1998). Meanwhile, there were also research indicating that the use of concept map can facilitate meaningful learning and be of value as a knowledge acquisition and sharing tool (Coffey et al. 2003). From the perspective of community-based learning, Fischer et al. (2002) found that by being provided with a content-specific visualization tool, both the process and out of the cooperative effort improved. Furthermore, collaborative concept mapping in a digital learning environment was also proved to be effective in overall learning gains and knowledge retention (Lin et al. 2012). As a result, the concept/knowledge mapping, as a visualization tool, has proved to be effective in both self-directed and community-based learning. For these reasons, in order to help those who constantly use the web for resource finding and organization, this research is setting off from the basis of visualizing the basic learning behavior of the learners such as searching for suitable information, organizing found learning information, and getting easier access to community-based well-organized learning resources through superimposed map representations. We target the open-ended learning resources on the web, with the purpose of providing learners with a user-friendly interface which intends to integrate self-directed learning into community-based learning.

### Research requirements

By analyzing these three difficulties described above and the contexts in which the self-directed learners regularly occur, we come up with three corresponding requirements, which if satisfied, could greatly enhance the current learning situation. These requirements are as follows:

1. More semantically structured representations for web resources in order to locate the candidates of learning resources more swiftly and correctly.
2. More sophisticated methods of resource organization. The learners often use web browsers for information management by simply adding interesting links to their favorite lists; however, this does not facilitate later learning activities such as reviewing to build knowledge structures. Here, one point needed to be stressed is that supporting learners with the process of building knowledge structure is not the focus of our research, as it requires considerations such as the attitudes, skills, and competences of the learners as well as reflection and self-construction which will be considered in our future work. We simply provide the learners with a meaningful structure of the learning resources as a visual aid for their knowledge building while reviewing the learning resources they have organized.
3. A visual space not only where the status of other learners’ resource collections can be explicitly represented but also where sharing resources and exchanging feedback can take place.

The following sections discuss how difficulties arising from the three requirements can be effectively addressed.

## Method

**Fig. 3:**
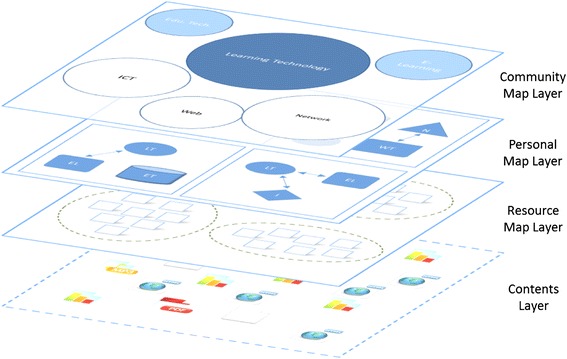
Multi-layer map model

Visualization is one of the keywords in this research, for its advantages of making complicated things seem simpler and easy to understand. As the purpose of this research is to visualize the basic learning behaviors of web-based self-directed learning, we proposed a model called Multi-layer Map Model aiming to realize basic learning behaviors on the web via map representation. The Multi-layer Map Model is the core of the proposed learning environment, which is intended to perform as a GUI for self-directed and community-based learning. Figure 3 shows the four layers of the model; each has different functions, yet is dependent on the services provided by their nearest layer. The contents layer is the lowest layer of this model, where actual web contents in various digital forms are located. The resource map layer is the place where the structure of the web contents is visualized as learning resources. The personal map layer is where the learners engage in their self-directed learning. They can define topics, build up connections between topics, and include the learning resources represented on the resource map layer in the topics they create. The community map layer merges the personal topic maps with those of other community members by displaying bubble charts based on their features and relations.

The model provides members of the community with a communication basis via superposed map representation. It primarily focuses on visualizing the structure of the learning contents in terms of a resource map and then enables the learners to edit or reconstruct their personal maps according to their learning processes. Moreover, this model includes a community map where the personal maps are merged, viewed, and used by other community members who have similar interests. This model is based on the concept of Topic Maps which is explained in the next subsection.

### Topic maps

Topic maps is an ISO standard for describing knowledge structures and associating them with information resources (ISO/IEC 13250 2002). The web enables us to create virtually unlimited quantities of information and to make it immediately available to the world. We do not suffer from lack of information availability, but we do suffer from finding the information we really need. Topic maps provide a standard approach to create and interchange finding aids (Park and Hunting 2002). While it is possible to represent immensely complex structures using topic maps, the basic concepts of the model—Topics, Associations, and Occurrences (TAO)—are easily grasped (Pepper 2000). Although by comparison, Wisse (2006) raised questions toward topic maps for its isolation resulted from unfamiliar wordings to members of the new information professions, we focus on its capability of representing complex structures in the context of learning which only involves learners with similar learning interests and goals.

**Fig. 4:**
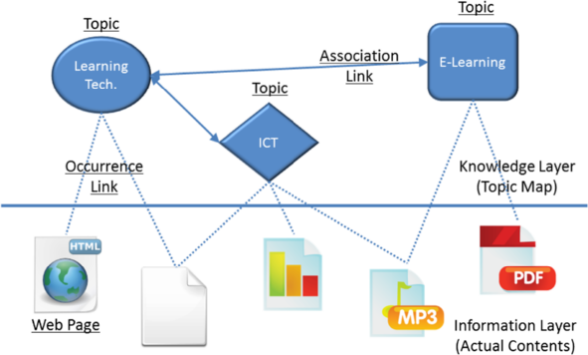
Basic concepts of topic maps

Figure 4 illustrates how the three basic concepts relate to the topic maps and how this ISO standard is applied to our research. Topics represent concepts of a certain field in which a learner is concerned. Association links represent hyper-graph relationships between the topics. Occurrence links represent the actual web contents relevant to a particular topic. In Fig. 4, there are three topics of the learners’ interests: Learning Technology, ICT, and E-learning. The solid lines among these three topics are associations which depict the various relationships the three topics have with each other. The dotted lines under these three topics are occurrences which represent the actual web contents in various digital forms. Topic maps can be used to qualify the contents and/or data contained in information objects as topics, to enable navigation tools and to link topics together with multiple, concurrent views on sets of information objects. A detailed discussion of how the concept of topic maps is applied in every layer of Multi-layer Map Model is held in the following.

### Contents layer and resource map layer

**Fig. 5:**
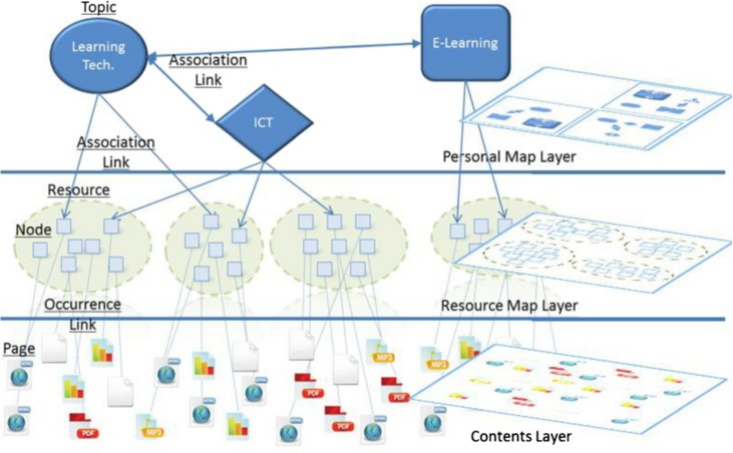
Relationship diagram among the layers of personal map, resource map, and contents

Contents layer is the lowest layer of this model. It means the actual web contents such as web pages, documents, and media files of the web-based learning resources. Resource map layer is the place to visualize structures of the web contents by a bunch of nodes in a one-to-one manner as shown in Fig. 5. This map is intended to provide the learners with an overall perspective of the learning resources which is expected to enable them to grasp the main content of web information more swiftly and precisely (Herman et al. 2000; Roto et al. 2006). Every node will be labeled with a typical word such as the title of the web page existed. The learning behaviors of searching for suitable learning resources and categorizing selected ones are conducted at this layer.

### Personal map layer

Personal map layer is aimed to support the learner’s self-directed learning. It helps the learners to edit and reconstruct their personal topic maps based on the spatial maps created on the resource map layer. At this layer, the learners are capable of defining the topics, adding/deleting the occurrence links under the certain topic, building up the association links among the topics, and navigating organized learning resources using the semantic structures of their personal topic maps.

### Community map layer

**Fig. 6:**
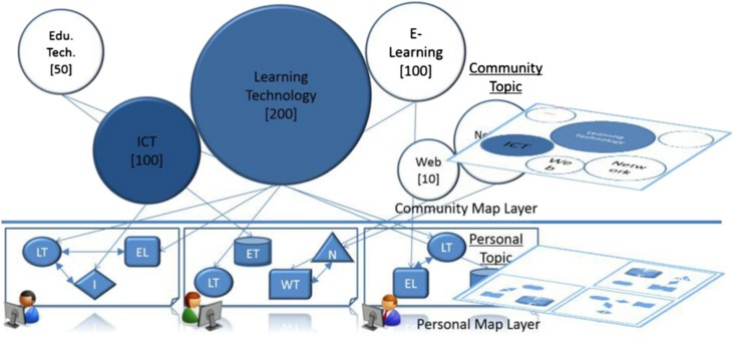
Concept of community map

For the purpose of sharing the learning resources in the community, community map layer merges the personal topic maps with that of other community members by displaying bubble form charts based on their features and relations as shown in Fig. 6. For the purpose of providing the learners sufficient information on community-based learning resources, the features of the bubble are containing useful information. The size of each bubble represents the number of occurrence links in a topic. The relative positions of the bubbles are calculated by the number of the association links among the topics, and the color of each bubble represents the relevancy to that of the learners’ learning topics. As the effectiveness of sharing and managing community-based knowledge through the application of knowledge map has been indicated in the related research (Lin and Hsueh 2005; Lin et al. 2006), in this research, all the topics and learning resources in the community will also be presented in map-oriented manner which is expected to enable the learners to locate and compare useful learning information more conveniently. The size and color of each bubble can be easily managed. However, the distance among the bubbles and the position of each bubble are difficult to calculate. As the bubbles represent the topics created by the learners, the distances among them are perceived as the level of relevancy among the topics. The closer the bubbles are, the more related the represented topics might be, which is expected to give the learners hints of priority for reference. In the next section, we introduce how to position the bubbles by adapting a spring model approach.

### Sequential spring model map for visualization of community map layer

In this section, we introduce how to visualize the topics as a concept map for the community by adapting the spring model approach sequentially (Hasegawa and Li 2012). This is expected to inform the learners of the relationships among the topics in terms of community map generated automatically, which has multi-dimensional input without explicit links.

#### General spring model algorithm

As the distances among the bubbles are affected by the ever-changing personal topic maps of each individual, the relevance among the topics is constantly changing all the time. Sometimes they might be closely related with each other and need to be brought nearer, but sometimes they might turn out to be less related and need to be brought further from each other. As a result, we adopted Eades’s (1984) spring model to satisfy this need. This model is based on force-directed graph drawing algorithms which are a class of algorithms for drawing graphs. It aims to position nodes of a graph by assigning forces among the set of edges and the set of nodes, based on their relative positions. In this spring model, spring-like attractive/repulsive forces based on Hooke’s law are used to attract pairs of endpoints of the graph’s edges toward each other, and by using related algorithms, the places for all the nodes can be decided. We believe that by using this method, maps with fewer number of nodes and edge lapping are possible to be generated in a higher speed. However, as there are no edges in the community map and the necessity of calculation time, we have made changes to the original method Eades proposed to meet the needs of this research.

#### Proposed arranging algorithm

By referring to a related research on sequentially applying the spring model for fast node arrangement, in this research, we firstly set the importance of each node, then take into account of *no explicit edge among the nodes*, and finally propose the arranging algorithm for the community map. As we used bubble form chart in the community map, we refer the nodes as bubbles in the following paragraphs.

##### Calculating the importance of each topic

First of all, as the quality of an object is an important factor in Hooke’s law and the need for assigning importance to each topic, we decide each bubble’s quality and size by using the following formula. The importance *W*_*I*_ of topic *i* in the community map can be calculated by the following formula.

1 $$ {W}_i={\displaystyle \sum {a}_j{\displaystyle \sum {C}_{i,j,k}}} $$

*C*_*i,j,k*_ is used to standardize parameter *j* which is related to the topic *i* created by a learner *k. j* represents the frequency of topic appearance and the number of web pages contained in the topic *i.* On the other hand, *α*_*j*_ indicates the weight which is set beforehand according to each parameter. This formula calculates the size and quality of every bubble in the community map, indicating the popularity and information volume of each topic.

##### Calculating the relevancy among topics

After deciding the quality of each bubble, we need to place the bubbles into proper position to show the relevancy among the topics. The nearer they are, the more related the two topics might be. The relevancy *R*_*m,n*_ among the topics can be calculated using the following formula.

2 $$ {R}_{m,n}={\displaystyle \sum {\beta}_l{d}_{l,m,n}} $$

In this formula, *d*_*l,m,n*_ is used to standardize parameter *l* which is related to the relevancy between topic *m* and *n. l* represents the types of parameters which could be perceived as the relevancy among the topics. It could be the number of web pages mutually contained in different topics and the number of the association links among topics. We use the number of association links among topic to indicate the relevancy. *β*_*l*_ stands for the weight set initially for each parameter. This formula is used to calculate the distance among the bubbles in the community map.

##### Setting the initial position for each bubble

Firstly, the bubble with the biggest importance value calculated by the formula 1 will be put in the center of the community map. Then the other bubbles will be placed sequentially according to their importance (which means from the second largest bubble), and the distances among all the bubbles are calculated by the formula 2. As to attain their exact positions, the following equations of motion are applied.

##### Approximate calculation of motion equations based on Euler’s method

Because there are no explicit edges among the bubbles in the community map, we suppose that all the bubbles are linked with invisible springs. By following Hooke’s law (3), the spring that has both ends attached to two bubbles, the free end is being pulled by a force that magnitude is *F*. Suppose that the spring has reached a state of equilibrium, where its length is not changing anymore. Let *X* be the amount by which the free end of the spring was displaced from its *relaxed* position (when it is not being stretched or compressed).

3 $$ F=KX $$

As a result, we have come up with the below equation with which the force moving bubble *m* and *n* can be calculated.

4 $$ {F}_{m,n}=-K\left({D}_{m,n}-{R}_{m,n}\right) $$

*K* is the spring coefficient, and *D*_*m,n*_ is the distance between bubble *m* and *n*, and *R*_*m,n*_ is the desired distance between *m* and *n* calculated by the formula 2. From the motion equation, we have learned as the following, we can deduce the equation to calculate the position of the bubble pulled or propelled by the force *F*.

5 $$ F=ma $$

6 $$ a=v\hbox{'} $$

7 $$ v=r\hbox{'} $$

8 $$ r"=F/m $$

Here, supposedly bubble *i* is pulled/repelled by the force *F*_*i*_, while the position of *i* is *X*_*i*_, and the quality of the bubble is *W*_*i*_. We can repeatedly use the following motion equation to decide the proper position of the bubble *i*.

9 $$ {W}_i{X}_i"={F}_i $$

However, because we do not need to complete a perfect physical simulation, we approximate the speed and position during Δ*t* for *t* + 1. According to the Euler method (Euler 1768) which calculates the approximate value of speed difference between the starting point and the end point of the time period Δ*t*, we can get the following formulas to get the value of speed at the time of *t* + 1, and the according position at that time.

10 $$ {V}_{t+1}={V}_t+\left({F}_t/{W}_t\cdot \varDelta t\right) $$

11 $$ {X}_{t+1}={X}_t\cdot \left({V}_t\cdot \varDelta t\right) $$

Moreover, in the actual calculation, to control the bubbles from overlapping and the non-stop motion, we adopt the frictions decided by the velocity of each bubble. When *R*_*m,n*_ reaches a certain point at which the velocity of the bubble is near to zero or on the verge of overlapping with others, the calculation stops.

## Resource organization system for self-directed and community-based learning

### System architecture

**Fig. 7:**
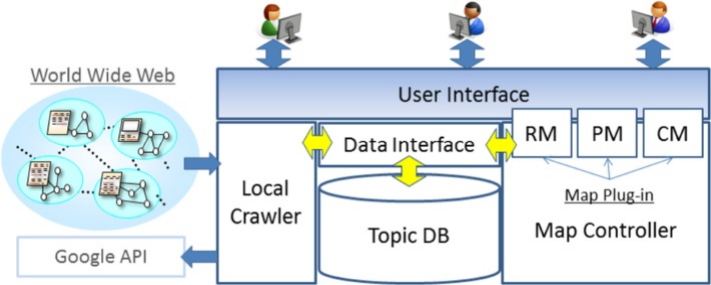
System architecture

Figure 7 shows a block diagram of the whole learning environment which contains main functions of the system. The learners are interacting with the system through the user interface where the three Map Plug-ins (RM, PM, and CM) are responsible for providing them with superimposed map representation. The local crawler is for collecting information from the web and storing the information in the form of Topic Maps (XTM) into the database through data interface which, at the same time, is also the channel for data communication with RM. Among all the functions in the system, two distinctive ones which are Local Crawler and Map Controller are worth to be discussed here.

The traditional search engines like Google is the first thing we can think of using when it comes to searching information. Therefore, in order to find related lists of URLs, it is necessary to embed some common search engine into this learning environment. As soon as the embedded search engine outputs a bunch of related URLs, the learners can select the link with the most relevance. Local crawler gathers the information of URLs of the web pages contained in the selected link and their titles, and then stores the gathered information to the database in the format of XML files according to the Topic Maps standard.

Map Controller is responsible for map editing and visualizing through layers of the resource, personal, and community map. As maps created at the upper three layers have their own features, each layer has their own map plug-ins. Resource map plug-in (RM) generates spatial maps automatically based on the results from the local crawler. It shows the structure of the crawled URLs in the form of nodes labeled with the titles representing the actual contents of the selected link. By clicking each node, the learners can access to the actual web page. Personal map plug-in (PM) drafts the personal topic map initially. The learners can edit their own personal topic maps by adding or deleting certain nodes, building association and occurrence links. Several association types are defined in the plug-in as super-sub (is-a), related terms, synonym, antonym, etc. Community map plug-in merges the personal maps created by community members and represents the maps with conclusive bubble form charts. The representation itself is expected to provide hints to the learners about the relevance of all the topics in the community with their own learning topics and information volume of all topics created.

### System overview

Based on the Multi-layer Map Model, we also developed a pilot system (resource organization system (ROS)) using Microsoft.Net and Silverlight which visualized the basic learning behaviors when searching for information on the web. ROS is a supporting tool designed to assist web-based self-directed learning. It visualizes the basic learning behaviors when learners searching and organizing learning information from the web, and at the same time, making it possible to collect well-organized learning resources from a learning community.

#### Interface of contents and resource map layer

**Fig. 8:**
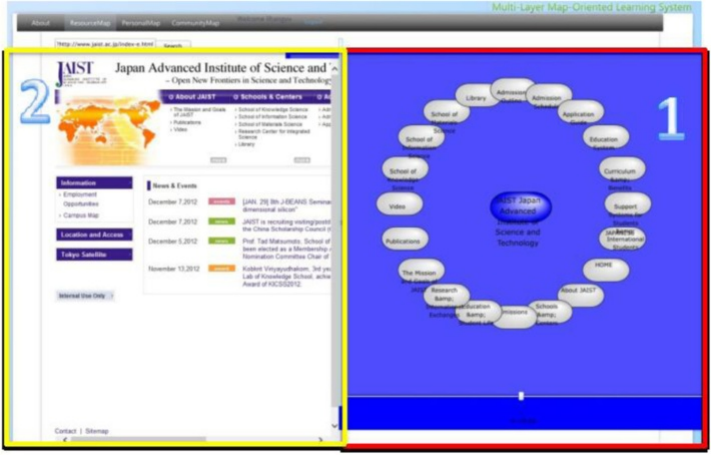
The interface of the contents and resource map layer

**Fig. 9:**
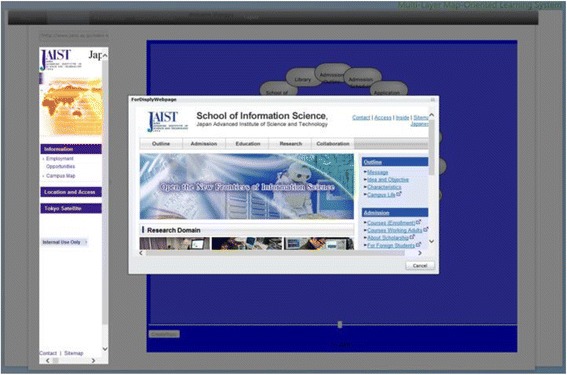
Viewing contents at resource map layer

The spatial map introduced by Kashihara et al. (2002) in their navigation planning system visualized all the web pages contained in one website in the form of nodes labeled with the titles. Gaines and Shaw (1995) took a different approach which generated one node at a time following the learners’ clicking activity on the web. We combined the both methods for generating the maps in our system. ROS not only provides the spatial map of the current selected links but also expands the spatial map generated interactively by the learners’ clicking activity. After logging into the ROS, the learners first use the embedded search engine API to select links most relevant to their interests from the web. The local crawler next gathers URLs and titles from the selected links. ROS subsequently generates the spatial map as a resource map automatically based on the results gathered by the local crawler. Figure 8 shows the interface of contents and resource map layer. On one side of the window (block 1), it shows both the structure of the selected Url in the form of nodes labeled with their page titles, and the actual web page of the selected link on the other side of the window (block 2). By checking the real web pages and their semantic representations at the same time, this arrangement is intended to increase the speed and accuracy of the learners’ comprehension of the main contents of the links. On the one hand, the learners can access the contents by clicking on a node as shown in Fig. 9 by a pop-up window where the web page of the selected node will display. While on the other hand, they can generate the corresponding resource map on the right correspondingly by clicking a link of the web page on the left.

**Fig. 10:**
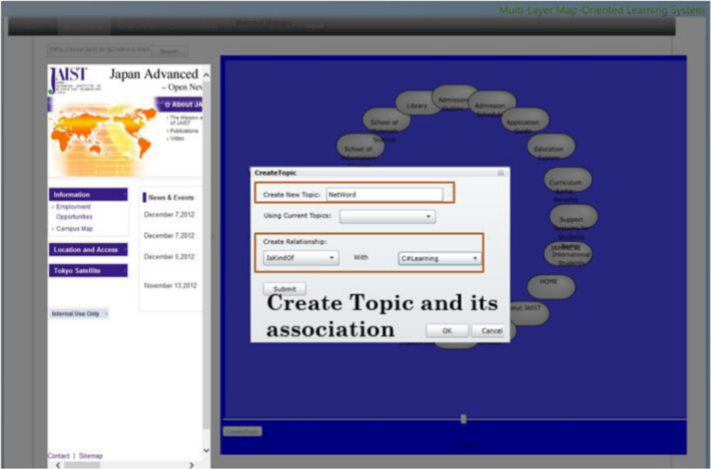
Creating topic and its association

**Fig. 11:**
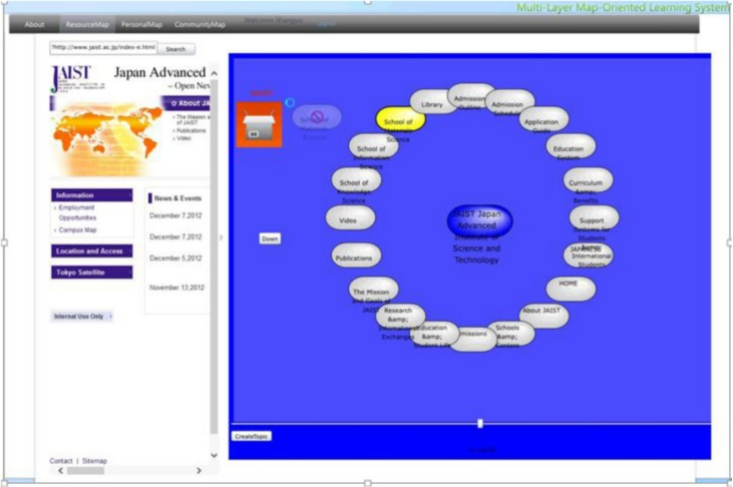
Store links by drag and drop

When the learners have viewed enough, it is time for them to organize the web pages interested in them through the creation of personal topic maps. As Fig. 10 shows, they can create new topics or use the existing ones and build the associations among the topics. When they have decided on the learning topic, a little icon will appear on the left upper corner of the right block symbolizing the current learning topic, and they can drag and drop the nodes selected into the icon indicating that the chosen web pages have been stored and categorized as shown in Fig. 11.

#### Interface of personal map layer

**Fig. 12:**
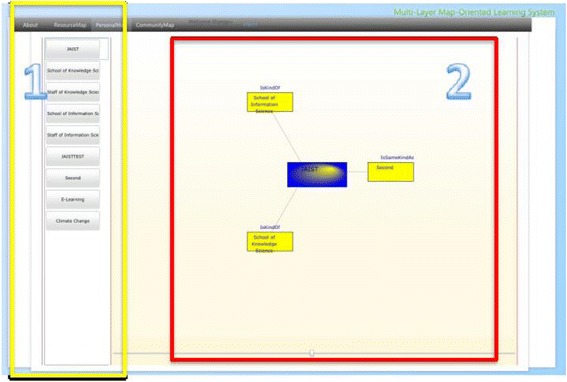
The interface of the personal map layer

**Fig. 13:**
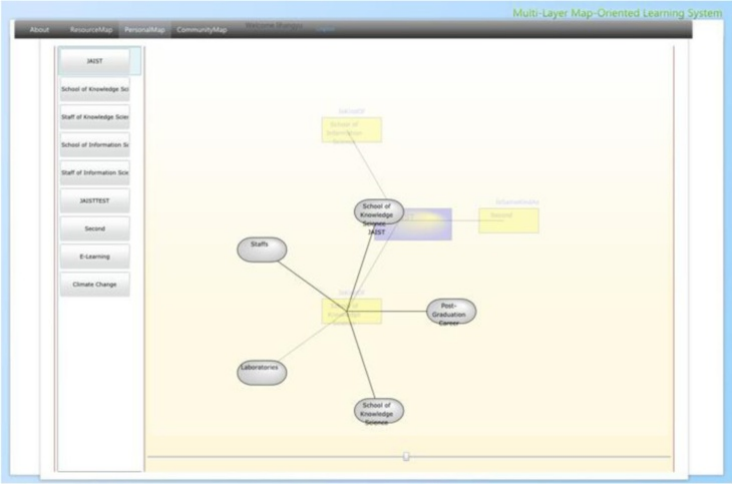
Viewing the content of personal map

Personal topic map in this research bears resemblances to the concept of knowledge maps/concept maps which have been frequently adopted in other learning systems. However, it is neither automatically generated (Chen and Xia 2009) nor created with the assistance of domain experts (Lin and Hsueh 2005). In the ROS, the learners’ conception of their learning goals and the learning resources prompt the creation of the topics which perform as both indexes and concepts/knowledge. The learners can view all the personal maps they have created as shown in Fig. 12. Block 1 shows all the learning topics one learner has created. By clicking one topic in block 1, the according personal topic map will appear in block 2 where not only the chosen topic will be shown in the middle but also the other topics related to the selected one and the types of the associations. By clicking into each topic in the personal topic map, the links the learner has stored in terms of nodes labeled with the link titles will appear as shown in Fig. 13. The learner can also check the contents of the corresponding web page by clicking into the selected node. The learners are expected to get to know the content of their chosen links by using this interface. The structures indicating relationships among the topics aim to provide the learners with an option of checking the contents of other related topics beside the chosen one.

#### Interface of community map layer

**Fig. 14:**
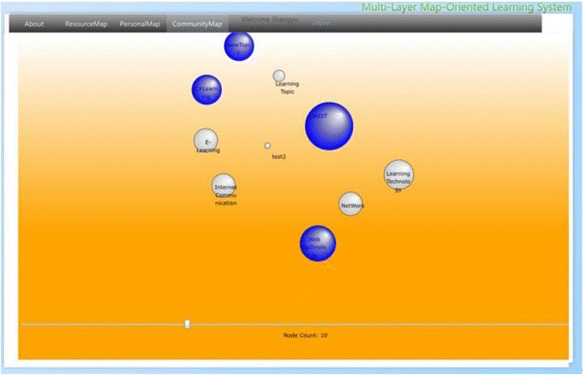
The interface of the community map layer

**Fig. 15:**
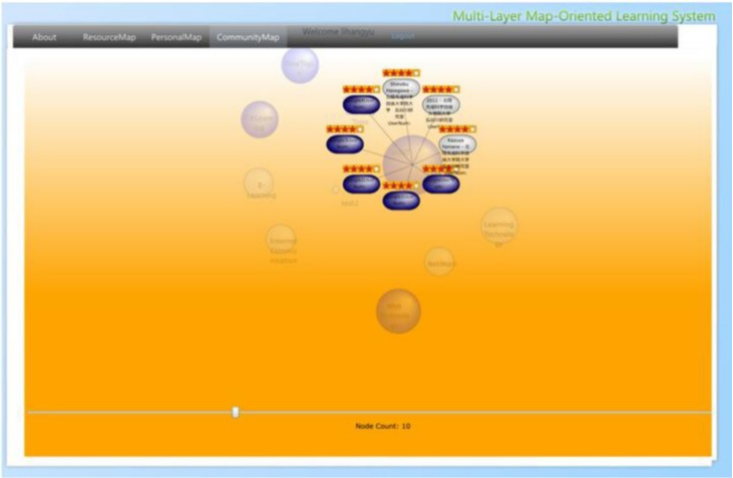
Viewing learning resources in community map

In a sense, community topic map in this research can also be taken as some sort of concept map. Unlike using concept map as a navigation tool in hypertext environments (Puntambekar and Stylianou 2003), or a means for measuring content understanding (Herl et al. 1999), we consider the community topic map of ROS as a conclusive presentation for community-based learning resources, combined with topics (concepts) existed among the learners of a learning domain. As shown in Fig. 14, ROS merges necessary information (number of learners under a same topic, number of learning resources under every topic, and the number of shared learning resources and associations among topics) of the personal topic maps and presents them in the form of a community topic map. Relevance to the topics of the current learner (colors of bubbles), relevancy among topics in the community topic map (distance between bubbles), and the number of learning resources under one topic (size of each bubble) give the learners hints for choosing learning resources of interest. We have applied the spring model discussed in the previous section for placing the bubbles which represent all the topics created in the learning community. After clicking a bubble, the learning resources will be presented in terms of nodes of a different shape labeled with their titles, which also can either be collected or ranked by the current learner as shown as in Fig. 15. As a result, the learners create their personal maps by referencing both the resource map and the community topic map. Learners’ personal topic maps contribute to the community topic map as well.

#### System flow

**Fig. 16:**
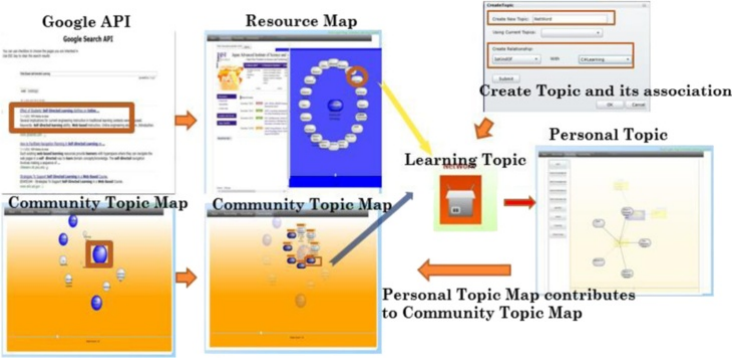
System flow

To sum up, at the beginning, the learners input keywords into Google API in order to get related search results so that they can look for the topics of interest at the content layer. If they select an interesting link from the search results, the local crawler gathers information of the web page selected and has it presented as resource map where they can create topics and drag and drop the selected nodes to the topics they have created. As community-based learning, the learners search some topics from the community map where all the topics and the according learning resources will be shown. They can also drag and drop the nodes under a certain topic and, at the same time, add new learning resources they have organized from the resource map. The system flow is shown in Fig. 16.

## Preliminary case study

In order to assess the effectiveness of this pilot system, especially by referencing the three requirements proposed, we conducted a quantitative case study followed by a qualitative one consisting of a questionnaire as an important component of this research. Sixteen graduate students participated in the case study. As the experimental environment (UI and experimental resources) is written in English, they are also required to have the similar level of English proficiency.

### Quantitative case study

Given that many self-directed learners are accustomed to using Microsoft Internet Explorer (IE) to search, organize, and learn information on the web, we designed a contrast evaluation plan in order to compare the advantages of using ROS versus IE for resource searching, organizing and sharing activities. From a series of preliminary experiment for making the rules for the official one, we designed the evaluation to be: the participants were required to use both IE and ROS respectively to conduct web-based self-directed learning on two different learning themes—E-learning and Environmental Protection from two websites (previously prepared, working as learning resources) within a fixed amount of time (30 min each) as shown in Table 1. For the control condition, 20 keywords working as subthemes were prepared for each learning theme. There were at least 10 web pages available to be checked on average for each subtheme, which makes a total of more than 200 web pages in each website, ensuring the participants’ impossibility to read through all pages within 30 min for the sake of control condition. One team containing four participants was required to complete their learning by using ROS or IE in different order to ensure adequate data samples were obtained (shown in Table 1). Since time for instruction for the participants and the refreshment time between phases was given, an extra 30 min were added to the experiment time, requiring a total of 1.5 h for a complete session. As a result, each participant was asked to conduct self-directed learning by using either IE or ROS under the themes of both E-learning and Environmental Protection within 1.5 h.

**Table 1:** The experiment arrangement

	Phase 1	Phase 2
Participant 1, 5, 9, 13	ROS(E-learning)	IE(PE)
Participant 2, 6, 10, 14	ROS(PE)	IE(E-learning)
Participant 3, 7, 11, 15	IE(E-learning)	ROS(PE)
Participant 4, 8, 12, 16	IE(PE)	ROS(E-learning)

#### Experiment procedures and evaluation factors

**Fig. 17:**
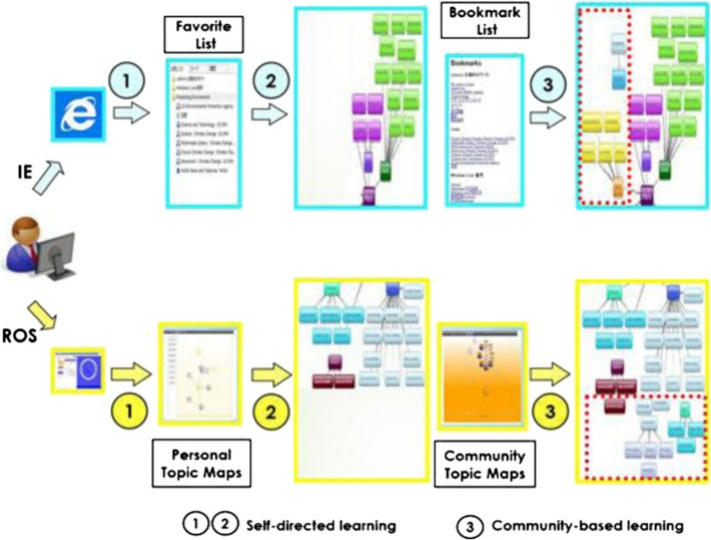
The experimental procedures and tasks

The learning goals for each participant were finding web pages and creating a knowledge structure based on the web pages found. The participants were first asked to find the web pages they considered appropriate from the two websites provided by using IE and ROS separately. In the case of IE, the pages found needed to be saved in the favorite list. In the case of ROS, by viewing the web pages and their generated resource maps simultaneously, the participants were asked to save the found pages in terms of personal topic maps by dragging and dropping the nodes to the topics created by themselves. Based on the contents stored in the IE favorite list or system’s personal topic map, the participants were asked to draw keyword maps on a paper; the keywords written were either extracted from stored content or created by the participants themselves while reviewing the web pages they had found. Here, we want to emphasize that those topics in the personal topic maps were created by the participants for categorizing found web pages and that the keywords written in keyword maps were those extracted or summarized from the web pages stored to describe the learning content. Finally, the participants were asked to review the web pages collected by the community members and add new keywords into the keyword map they had drawn previously. Here, as a control condition, we previously prepared two resource bases of community-based learning. All the subthemes were covered in the two bases, and each of them contained averagely 10 web pages which were all from the two previously prepared websites, making it impossible for the participants to read though all the contents within the time of community-based learning. As a result, only the situation of using community topic maps was evaluated in this experiment, not the function for generating community topic maps, which will be considered in a future study. In IE, the pre-prepared community-based learning resources were represented in terms of bookmark lists, and in ROS they were represented in terms of community topic maps. In summary, the participants were asked to conduct three procedures for the learning of the two themes respectively while using IE or ROS. The three procedures are as follows: “Finding learning resources (procedure 1)”→Drawing keyword map (procedure 2)→Supplementing keyword map (procedure 3)” as vividly shown in Fig. 17, there were evaluation factors indicating the learning effectiveness of the corresponding processes for each of these procedures.

Number of web pages found in procedure 1: this evaluation factor was chosen based on the first requirement listed in our research requirements. The semantic representations of the resource map offered by ROS are supposed to help the participants more swiftly and accurately locate potential learning resources, and the number of web pages found in a fixed time can best illustrate the efficiency of doing so.

Number of keywords drawn and web pages viewed in procedure 2: the second research requirement suggests that the learners need a more sophisticated way to organize and review found learning resources than using the favorites list of a web browser. The personal topic maps in ROS provide the participants with a more semantic management and a representation of learning resources, which are intended to facilitate later review. Therefore, the number of keywords drawn by reviewing the found resources is believed not only to filter out the irrelevant pages accidentally stored due to the rush, but also to evaluate the accessibility of the found learning resources represented by the ROS’s personal topic map. Moreover, by counting the number of web pages viewed from which the keywords were written, we can evaluate the efficiency of reviewing found web pages when using IE or ROS. One point that needs to be stated is that it must be the number of pages from which keywords are drawn, not those viewed without keywords having been extracted.

Number of keywords added and web pages viewed in procedure 3: based on the third research requirement, we designed the third procedure as community-based learning. The community topic maps in ROS give the participants overviews of the status of resource collections of other learners and the ratings (number of stars) as feedback for each learning resource. We considered the number of keywords newly added into the keyword map created previously and the web pages viewed for writing these new keywords valuable evaluation factors, in evaluating the efficiencies for resource sharing and searching in a learning community via map representation.

Number of keyword islands drawn within the keyword map eventually: this evaluation factor was not initially considered. However, when viewing the keyword maps drawn by all the participants, we found that the number of keyword islands (cluster of keywords) by using IE and ROS was very different. This might best describe the difference between the knowledge structures generated while using IE or ROS.

#### Results and discussion

Details are shown in Table 2. From the average data itself, we can easily see the difference in the use of IE and ROS in each group of data. However, we used a *T*-test to determine whether the means of the two groups were statistically different from each other and to assess whether the difference was meaningful or not. We can easily see from this table that *t critical two-tail* < |T stat.| and *p* < 0.01 from every group of data indicated that differences within each group were statistically significant.

**Table 2:** Experiment data with *T*-test

	Ave. (ROS)	Ave. (IE)	T stat	*T* critical two-tail	*P*(*T* ≤ *t*) two-tail
Web pages found	64	17.875	19.654	2.131	4.06E-12
Keywords drawn/pages viewed	48.312/14.437	21.6875/6.75	10.052/11.181	2.131/2.131	4.67E-08/1.13E-08
Keywords added/pages viewed	35.437/12.5	16.1875/6.5625	7.066/6.188	2.131/2.131	3.83E-06/1.74E-05
Islands	1.812	4.8125	−7.745	2.131	1.28E-6

In this experiment, we evaluated the effectiveness of using ROS for the participants in their web-based self-directed learning combined with community-based learning. Before getting into the discussion of the experimental results, we need to address that although we have evaluated the community-related function which is using the community topic map of the ROS to support the participants’ self-directed learning in resource searching and organization in a learning community, we did not examine the effectiveness of community-based learning which requires further evaluation of the process for generating community topic map. In this case study, we only used determined expert data for condition control. In the future, we will take account of this factor to evaluate how the creation of community topic map affects community-based learning.

**Fig. 18:**
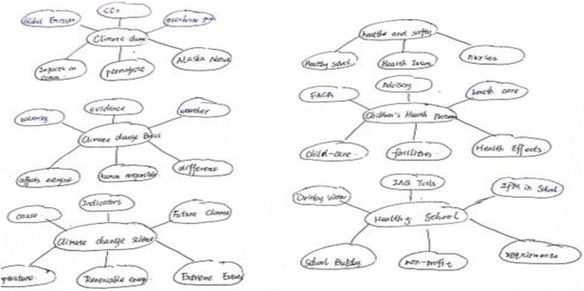
Example of keyword map when using IE

**Fig. 19:**
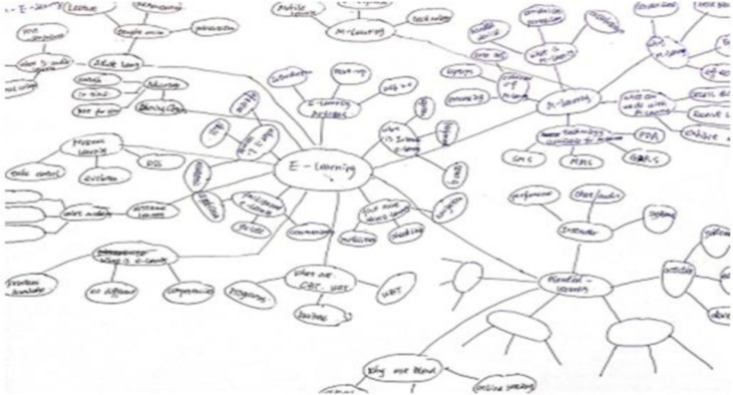
Example of keyword map when using ROS

Based on the results of the data analysis, the following conclusions have been drawn:

- ROS enables the participants to find more web pages. This conclusion indicates that the visualization of the explicit structure of selected links and enhanced semantic representation of its contents on the resource map of ROS enabled them to overcome the complexity and obtain learning resources they thought appropriate to their learning goals faster and more correctly.
- ROS enables the participants to write more keywords from more web pages viewed. Due to the limitations of organizing information using browser’s favorite lists, ROS simplified the process by enabling them to create personal topic maps, to which interesting web pages (occurrences) were added and relationships among topics (associations) were built. The data suggest that, due to its easy accessibility and meaningful structure, the personal topic map of ROS played a positive role in the process of reviewing the learning resources.
- ROS enables the participants to write more keywords from more web pages viewed in community-based learning. The community topic map of ROS gave the participants overviews of all the learning topics and the learning resources of their learning community, which enabled them to quickly locate the necessary learning resources, and because of which, as the result indicated, more keywords had been written.
- ROS enables the participants to draw less keyword islands eventually. This result was unexpected and thus had not been considered as an evaluation factor at the outset. However, when examining keyword maps drawn by every participant in aggregate, we found that the number of keyword islands was 62 % less when using ROS than that of using IE, as shown in Figs. 18 and 19. Not only that, the average number of keywords (drawn in procedure 2) in every keyword island created using ROS was 26.66, greater than that of keyword islands created using IE which was only 4.50. There were relatively few connections among main keywords in the drawings created by IE users; however, when the meanings of most keywords were considered, it seemed reasonable to think that connections should have been made. Comparatively, ROS users performed well as indicated by the number of connections that had been drawn and the number of keywords added. This change, after consulting each participant about the reason those connections were being made, is due to the structure of personal topic maps where the basic connections (associations among the topics) were already present. They were conducting self-directed learning with the awareness of the connections among topics; therefore, the connections were made among keywords extracted in their learning. Take the example created by one participant (as shown in Fig. 19) for instance: in his/her personal topic map in ROS, there were topics of E-learning, Adult learning, M-learning, and Distance learning. E-learning seems to be the main topic, and the others seem to be the topics related to it. We can see these connections among these topics in his/her keyword map, and the keywords around these topics were extracted from web pages stored in these topics in his/her personal topic map. This accidental finding indicates that semantically structured representation of learning resources can give the learners positive impact while reviewing their learning materials for knowledge construction.

### Qualitative case study

Followed by the quantitative case study, we also conducted a qualitative one requesting each participant to fill a questionnaire after the quantitative experiment. The questionnaire was designed to investigate the participants’ thoughts on their use of ROS and IE during their tasks and the reasons for their performance. Furthermore, their customs of searching and organizing learning resources on the web were asked to further address our research purposes. Meanwhile, their expectations on the improvement of system functions were also inquired in order to collect practical suggestions on future system development to ensure user acceptance.

#### Questionnaire content

Table 3 shows all the questions in the questionnaire. Q1 to Q5 were designed to ask the participants’ thoughts on their use of ROS and IE during their tasks and the reasons for their performance. Especially, Q5 was aimed to reflect their future acceptance of the learning support system like ROS, which was intended to make us evaluate our research from the practical aspect. Q6 and Q7 were simply to investigate the participants learning habit when it comes to using IE or other browsers for resource searching and organization, basically to grasp the learning situations for web-based self-directed learners. Q8 was mainly to collect the participants’ practical suggestions on system improvement, which will be taken into account for our future development and remedy of the system, aiming to ensure user acceptance of the system developed.

**Table 3:** Questionnaire

	Content	Items
Q1	Which functions were more helpful for you in searching for web pages related?	A. Strongly ROS;
		B. ROS;
		C. Mildly ROS;
		D. Similar;
		E. Mildly IE;
		F. IE;
		G. Strongly IE;
		Reasons for the choice:_____
Q2	Which functions were more helpful for you for saving the links you find useful?	Same as above
Q3	Which functions were more helpful for you when reading pages for keyword drawing?	Same as above
Q4	Which functions were more helpful for you when reading pages in community-based learning for adding keywords to your keyword map?	Same as above
Q5	Are you willing to use ROS for searching and organizing web pages for self-directed learning?	A. Strongly yes;
		B. Yes;
		C. No;
		D. Strongly no;
Q6	Did you always save the links you find useful into IE favorite list?	A. Yes;
		B. Sometimes Yes;
		C. Sometimes No;
		D. No;
Q7	Did you always categorize the links you found in your IE favorite list?	A. Yes;
		B. Sometimes Yes; C. Sometimes No;
		D. No;
Q8	What are your suggestions for the improvement of ROS in the future?	A. About resource map:____
		B. About personal map:____
		C. About community map:__
		D. Others:_______________

#### Results and discussion

From the results of the questionnaire as shown in Table 4, we have drawn the following conclusions:

**Table 4:** Result of questionnaire (item/number of participants)

	A	B	C	D	E	F	G
Q1	7	7	2	0	0	0	0
Q2	6	9	1	0	0	0	0
Q3	1	11	3	1	0	0	0
Q4	5	4	7	0	0	0	0
Q5	6	5	5	0			
Q6	1	5	10	0			
Q7	1	0	10	5

- From Q1 to Q4 which were asked to evaluate the usefulness of ROS for executing the learning tasks regarding the requirements as described in the previous section, we concluded: Firstly, all participants considered ROS more helpful for their searching for related web pages. According to the reasons written down by some of them, we can conclude that the ROS’s resource map was playing a positive role in this procedure, and the two screens for displaying resource map and the actual web page, pointed out by 3 participants, were helpful also. Secondly, all participants consider ROS more helpful when saving the links they found useful. Some participants noted that it was due to the easy operation of dragging and dropping nodes from resource map that facilitated the number of links stored using ROS surpassed that of using IE. Meanwhile, as a participant pointed out that the compulsive operation of creating topics and building connections among them made their search more targeted. Thirdly, 15 participants considered ROS more helpful when reading pages for keyword drawing. The reasons for this choice, according to some participants’ comments, were for the structure of learning topics whose connections were built by themselves previously presented by ROS’s personal topic maps. As a participant stated: “When I looked at the personal topic maps, I can recall the reasons for adding these learning resources to the topics and also be reminded of the relationships among all the learning topics I had created. This helped a lot when trying to figure out the contents of the web pages, making the drawing keyword map easier.” However, only one participant found it similar whether using ROS or IE, the reason for this was that he/she did not find it more convenient reading pages from personal topic maps than IE’s favorite list as both needed them to selectively read through for keywords. Finally, all participants considered ROS more helpful when reading pages in community-based learning resources for adding keywords to their keyword maps. For those who had written down the reasons for this choice, they attributed their better performance using ROS to the clearer representation of topics and learning resources of the community map.
- The results of Q5 indicated that 11 participants were willing to use ROS for searching and organizing web pages for their self-directed learning based on their experiences in the case study. However, there were still 5 participants who clearly expressed their unwillingness toward the idea of using ROS for future resource searching and organization. They explained that it was true that using ROS proved to be better to perform the learning tasks designed in the experiment, but the ROS’s supporting functions were not convenient enough to replace IE or the likes which they had been accustomed to use. The reasons were revealed in Q8 of the questionnaire.
- From the results of Q6 and Q7, we can see that most participants (10/15) seemed that they seldom saved the links they considered useful to the favorite list of IE or other browsers they might be accustomed to use. Moreover, it also showed that most participants did not have the habit of categorizing the web pages they stored in the favorite lists. By mandatorily making the participants create topics and build relationships among the topics, ROS can improve learners’ awareness for saving and organizing the learning resources found on one hand but has the possibility of causing hesitations and anxieties in the learners having not decided on the topics and associations. We will add more flexibility in the future.
- Finally, from the comments on the future improvement of ROS, we received several practical advices related to the changes and expected functions on the three map representations. As to the resource map, they thought it would be better to show more information on the map besides nodes and page titles; some suggested that it would be better if the system would recommend some related links by lightening up certain nodes. Some pointed out that it was necessary to provide the learners with the option of *dig deeper* into the links selected with more layers of nodes other than just one layer. As to the personal map, they wanted more supporting functions to take more actions such as taking node, viewing the whole picture of all the topics created and their relations, editing the content of the web pages by adding or trimming particular parts, and the option of viewing other learners personal topic maps. For community map, some pointed out that it was better if they were able to evaluate the learning resources by typing text messages besides using star icon, and if the system could recommend some related learning resources to them before getting started on viewing all the resources. We will take these suggestions into consideration and resolve to reflect them in our future development of the system.

## Conclusions

This research proposed a Multi-layer Map Model by employing the methodology of topic maps to address several difficulties in web-based self-directed learning. We also developed a resource organization system by using Microsoft.Net and Silverlight which enabled the visualization of the basic learning behaviors of searching for and organizing information from the web. Based on the results of the case study presented, we are able to conclude that the learners using the proposed model performed better on tasks that required them to locate and organize learning resources. We can also tentatively state that building connections among learning topics not only provides a better means of resource management but also is subconsciously helpful in the creation of knowledge structures. And the qualitative study further addressed that ROS helped the participants in every aspect during their execution of the learning tasks.

In the future, we will improve the current model’s functionality by introducing another ISO standard (ISO/IEC 19788 2002) which is to use metadata for better descriptions and retrieval of learning resources besides the web page title both in self-directed and community-based learning, enable the learners not only to categorize the learning resources they found on the web, but also to locate their needed learning resources in the learning community. We also want to focus more closely on community-based learning (CBL). The community here means a group of people sharing similar learning interests but with different knowledge levels and learning goals. Such diversity inside the community makes interaction among community members possible; if such interactions could be better utilized and community knowledge or skill shared and inherited, each individual’s learning activity can be expected to improve. However, the current learning environment does not enable the learners to take complete advantages of CBL activities, as communications cannot be passed promptly, advanced learning skills cannot be properly observed, and community-level knowledge structure is difficult to recognize. Combined with the results of current research, we want to emphasize more on factors of CBL, which is expected to play an important role in people’s learning activities.
